# First Observation of Hb South Florida [beta 1(NA1) Val>Met] in Turkey

**DOI:** 10.4274/Tjh.2013.0014

**Published:** 2013-06-05

**Authors:** Ayça Dilruba Aslanger, Aynur Akbulut, Gül Tokgöz, Sakine Türkmen, Kanay Yararbaş

**Affiliations:** 1 Kocaeli Derince Training and Research Hospital, Medical Genetics Department, Kocaeli, Turkey; 2 Kocaeli Derince Training and Research Hospital, Department of Pediatrics, Kocaeli, Turkey; 3 Kocaeli Derince Training and Research Hospital, Department of Hematology, Kocaeli, Turkey; 4 Hemoglobinopathy Screening Center, Child, and Family Planning, Department of Health of Mother, Kocaeli, Turkey; 5 Düzen Laboratory Group, İstanbul, Turkey

**Keywords:** abnormal hemoglobin, Hemoglobinopathy, HbA1c, Hb South Florida

## TO THE EDITOR

**Figure 1:**
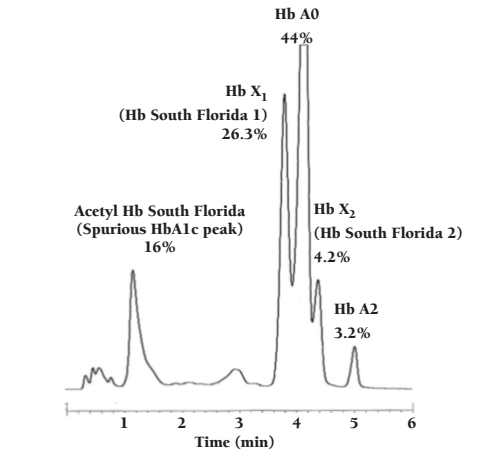
HPLC result of heterozygous Hb South Florida case.

Hemoglobin (Hb) South Florida [beta1(NA1) Val>Met] is a rare beta hemoglobin variant that was first reported in 1985 from South Florida [1,2]. We report here, for the first time in Turkey, a 17-year-old female originally from Kars with Hb South Florida. She was referred to the Hemoglobinopathy Screening Center because of the history of thalassemia in some of her distant relatives. The variant was detected by high-performance liquid chromatography (HPLC) and confirmed with DNA sequencing. HPLC was performed with a Primus Ultra2 Hb variant analyzer (Trinity Biotech Dublin, Ireland) with the following results: Hb rate for HbA0 44%, HbA2 3.2%, spurious HbA1c peak (Acetyl Hb South Florida) 16%, Hb X1 (Hb South Florida 1) 26.3%, Hb X2 (Hb South Florida 2) 4.2%, and HbA1 (the sum of all HbA1 forms including those with several minor peaks [range: 0.1%-3.4%]) 6.3% (Figure 1). Red blood cell parameters were all in normal ranges, as follows: Hb 13.2 g/dL, Hct 38.5%, RBC 4.8×1012/L, MCV 81 fL, MCH 27.7 pg, and MCHC 34.2 g/dL. HbA1c was shown to be 3.0% with the Glycohemoglobin Analyzer’s standard analysis mode (Tosoh Bioscience, Tokyo, Japan). DNA sequencing (ABI 3130 Genetic Analyzer, Applied Biosystems, Foster City, CA, USA) confirmed this Hb variant with a heterozygous mutation at the β-globin gene exon 1 codon 2 (c.4G>A, p.Val2Met, rs33395835, NG_000007.3). This mutation, next to the initiation codon, was named as codon 1 by the Huisman Database [beta1(NA1) Val>Met] [3]. 

Hb South Florida was first reported in a Caucasian boy who had markedly elevated HbA1c (14.8%) [1,2]. The detecting methods for HbA1c have the potential of detecting coexisting Hb variants or hemoglobinopathies. However, the HPLC techniques for HbA1c testing used in many hospitals are not designed for diagnosing Hb variants [4]. Thus, our case had a spuriously high HbA1c result of 16% in HPLC for variant hemoglobinopathy analysis mode, which measures acetyl Hb, and HbA1c of 3% in HPLC for standard analysis mode. Therefore, it is hard to identify this variant with a routine HbA1c measurement. 

This hemoglobin variant in the heterozygous state does not produce any clinical symptoms. Only one other patient from Malaysia has been reported since the original report with a compound heterozygote mutation of c.4G>A (GTG>ATG) and IVS1-1 (G→A) [5,6]. Although the combination of Hb South Florida with β-thalassemia was found in the Malay with no associated clinical symptoms except hematological results consistent with the beta thalassemia trait, we knew that the interactions between 2 different Hb variants could result in more severe disease. This is especially important for countries like Turkey where the prevalence of the β-thalassemia carrier state and abnormal Hb is very high [7].
